# Supporting Beacon and Event-Driven Messages in Vehicular Platoons through Token-Based Strategies

**DOI:** 10.3390/s18040955

**Published:** 2018-03-23

**Authors:** Ali Balador, Elisabeth Uhlemann, Carlos T. Calafate, Juan-Carlos Cano

**Affiliations:** 1 Innovation, Design and Technology (IDT), Mälardalen University, 72123 Västerås, Sweden; elisabeth.uhlemann@mdh.se; 2 RISE SICS Västerås, Stora gatan 36, 722 12 Västerås, Sweden; 3 Department of Computer Engineering (DISCA), Universitat Politècnica de València, 46022 València, Spain; calafate@disca.upv.es (C.T.C.); jucano@disca.upv.es (J.-C.C.)

**Keywords:** intelligent transportation system, vehicular ad hoc networks, MAC layer, token passing, IEEE 802.11p, beacon broadcasting, platooning, reliability, event-driven messages, safety applications

## Abstract

Timely and reliable inter-vehicle communications is a critical requirement to support traffic safety applications, such as vehicle platooning. Furthermore, low-delay communications allow the platoon to react quickly to unexpected events. In this scope, having a predictable and highly effective medium access control (MAC) method is of utmost importance. However, the currently available IEEE 802.11p technology is unable to adequately address these challenges. In this paper, we propose a MAC method especially adapted to platoons, able to transmit beacons within the required time constraints, but with a higher reliability level than IEEE 802.11p, while concurrently enabling efficient dissemination of event-driven messages. The protocol circulates the token within the platoon not in a round-robin fashion, but based on beacon data age, i.e., the time that has passed since the previous collection of status information, thereby automatically offering repeated beacon transmission opportunities for increased reliability. In addition, we propose three different methods for supporting event-driven messages co-existing with beacons. Analysis and simulation results in single and multi-hop scenarios showed that, by providing non-competitive channel access and frequent retransmission opportunities, our protocol can offer beacon delivery within one beacon generation interval while fulfilling the requirements on low-delay dissemination of event-driven messages for traffic safety applications.

## 1. Introduction

Safety, comfort and efficiency of both roads and vehicles have improved considerably over the last decade. Advances in wireless technologies, along with improved sensing and computational capabilities, are now paving the way towards fully autonomous driving. While a comprehensive implementation of autonomous vehicles on all our roads still remains somewhat futuristic, the introduction of Cooperative Intelligent Transport Systems (C-ITS), where vehicle awareness concerning its current traffic context is aided by information exchange with surrounding vehicles or near-by roadside units, lies just around the corner. Status updates about a vehicle’s position and speed, or other in-vehicle sensor data, can be periodically broadcasted within a whole area to provide input to a multitude of safety and efficiency applications such as intersection collision warnings or overtaking warnings. Current standardization efforts focus on protocols for this short range inter-vehicle safety information exchange, and a dedicated frequency band in the 5.9 GHz range has been reserved in several countries for this purpose. At the same time, C-ITS systems will also require event-driven messages triggered by unforeseen events (e.g., a sudden deceleration through sudden breaking). Both periodic status updates and event-driven messages carry highly time-critical data, which imposes high demands on the timing and reliability of the underlying communication protocols.

An application enabled by C-ITS technology that has received much attention within the research community, as well as by the vehicle manufacturing industry and governmental organizations, is platooning of (heavy) vehicles. Consider a platoon of tightly spaced vehicles driving on a busy highway. The leading vehicle is operated by a driver, while all following vehicles are operated autonomously once their drivers have joined the platoon, and activated the platooning mode. Several studies have shown considerable reductions in fuel consumption by vehicles driving in close proximity in a single lane. In [1], Bonnet and Fritz could show a 21% fuel reduction for trailing trucks traveling at 80 km/h, and for an inter-vehicle gap of 10 m. Even the lead truck showed a fuel reduction of 6%. With 5% of the total global carbon emissions accounted for heavy vehicles, the large environmental benefits become a clear incentive for the transport sector.

Driving at highly reduced inter-vehicle distances requires a fully automated operation of the platoon vehicles (with the exception of the platoon leader). In-vehicle sensors, e.g., Lidar sensors to assess the distance to the vehicle in front, are not sufficient for the safe operation of the platoon at the targeted speed and gap sizes. C-ITS technologies can be seen as a set of adaptive sensors that provides additional information in order to constantly keep each vehicle up-to-date with the status of all other platoon members, and to inform about required changes to the current platoon configuration with minimum delay. The Medium Access Control (MAC) method of the recently standardized IEEE 802.11p (We recognize that 802.11p has now been integrated into the IEEE 802.11-2012, but use the term here to denote both the European variant ITS-G5 and the US variant DSRC of the former “p”-amendment to IEEE 802.11.) protocol suit for vehicular networking is based on the Carrier Sense Multiple Access (CSMA) algorithm. As shown in several papers published in the area, e.g., [2,3,4], CSMA is not able to provide guaranteed delay bounds with sufficient reliability for vehicular scenarios, especially under high channel usage. This problem is particularly serious when implementing a (semi-) automated driving application such as platooning, where inter-vehicle spacing is drastically reduced and the control loop that manages and maintains the platoon requires a frequent, timely and reliable exchange of status information (beacons). We, therefore, identified a need for a flexible and decentralized channel access protocol that considers the special properties and requirements of the platooning application, improves timely and reliable data delivery within the platoon and, at the same time, is easily implemented on top of 802.11p-enabled hardware.

In this paper, we consider a token passing protocol for safety data exchange within the platoon. While previous work [5,6] introduced the token passing method for periodic beacon exchange only, this paper extends the protocol to incorporate event-driven messages. We propose and evaluate three different methods to integrate event-driven data into our protocol, and we also consider the issue of relaying event-driven packets to improve the successful reception of such packets throughout the platoon. According to results from the CAMPANION project [7], an EU project on truck platooning, which was ended in 2016, we assume that the control channel is used for exchanging safety data with vehicles outside the platoon, and a separate service channel reserved to exchange platoon-specific information, such as controlling and directing information. Service channels can be used for certain applications such as platooning, as long as mandatory listening periods on the control channel are kept. Alternatively, a second transceiver can be installed and tuned to the service channel. Since heavy vehicles benefit greatly from platooning in terms of reduced fuel consumption, the benefits outweigh the extra cost of an additional transceiver. This separation enables us to deviate from the standard restrictions on, e.g., beacon update rates, message types and channel access method, to focus on the timely and reliable delivery of platoon control data.

The rest of this paper is organized as follows: in Section 2, we briefly explain the basic principles of the IEEE 802.11p MAC protocol and, in order to motivate this paper, we review the work related to the design of MAC protocols for platooning applications. In Section 3, we present our proposed approach in detail, clearly identifying the contributions of this paper compared with previous works. Section 4 provides an analytic evaluation of our protocol, whereas Section 5 describes the simulation scenarios and the selected metrics for performance evaluation. Simulation results are discussed in Section 6, and, finally, Section 7 concludes the paper.

## 2. Background and Related Works

IEEE 802.11p [8], an amendment to the IEEE 802.11 standard for inter-vehicle communications, defines specifications for the physical and MAC layers. Despite the built-in mechanisms of the CSMA MAC protocol to prevent packet collisions, such as listen-before-talk and back-off mechanisms, packets might still collide and lead to unbounded channel access delays, especially under heavily loaded channel conditions [2,4,9]. Results of many experiments show that the platooning application requires a beacon update frequency rate higher than 10 Hz, which is used as the common frequency rate for safety-critical applications [7]. In addition, when safety messages are transmitted in broadcast mode, no ACK message or RTS/CTS (Request to Send / Clear to Send) are transmitted to ensure a successful packet reception at the receiver side. Therefore, the IEEE 802.11p MAC protocol is unable to meet the delay and reliability requirements of platooning applications.

Recently, the European Telecommunications Standards Institute Technical Committee for Intelligent Transport Systems (ETSI TC ITS) changed its strategy of periodic generation of status updates, and instead using triggering rules for beacon message generation in order to mitigate the unnecessary load on the common control channel. According to these rules, a beacon message will be triggered based on vehicle movements, with triggering intervals between 100 and 1000 ms. Consequently, beacon messages can no longer be expected to be periodically generated. Therefore, it makes beacons in its current form unsuitable for highly safety-critical control application such as platooning, where it becomes vital that the control loop is periodically fed with fresh data.

Several MAC protocols have been proposed in an attempt to improve the communications delay and reliability of the standard, e.g., [3,10,11]. Although these proposals seek to keep channel access delay and packet loss at acceptable levels, they are designed to obtain benefits for generic Vehicular Ad Hoc Networks (VANETs), not tailored towards the specific requirements of a platooning application. Some works, however, do focus on practical issues and their impact on platooning performance, such as [12,13]. In [12], the impact of packet loss on the performance of platooning applications was evaluated, and, in [13], antenna placement and its impact on the packet error rate was evaluated using real-world experiments. Specific strategies to improve timing and reliability in platooning have been considered in the literature. In [14], the authors suggested two different technologies: IEEE 802.11p for the event-driven type of messages, and infrared (IR) for beacon broadcasting to improve reliability. Böhm et al. studied the co-existence of beacon and event-driven messages, showing how the choice of different MAC layer priority classes for beacon and event-driven messages, and also an adequate dissemination strategy for event-driven messages, can avoid overloading the medium with unnecessary data traffic and improve performance [4]. In [15,16], the authors introduced a general communications framework for centralized channel access having retransmission capabilities for safety-critical inter-platoon communications that was based on the data age of previously received messages. In addition, they argue that the service channel should be used for intra-platoon communications to provide the required level of reliability. Segata et al. showed that a combination of slotted scheduling and transmit power control mechanisms can improve reliability for platooning scenarios [9].

Time Division Multiple Access (TDMA)-based schemes have attracted an increasing amount of attention from the research community to provide benefits as follows [17]: high reliability, deterministic access time, efficient channel utilization, and equal access to the channel for all vehicles. For example, Fernandes and Nunes [18] analyzed five different TDMA-based MAC protocols to improve reliability for platooning scenarios. They suggest the use of priority levels and anticipatory information from the platoon members to improve the reactivity to, e.g., velocity changes. Moreover, Aslam et al. used Reconfigurable and Adaptive TDMA (RA-TDMA), which sets an overlay TDMA protocol on top of IEEE 802.11p to support concurrent collaborative applications in VANETs, including platooning application. The protocol is distributed and does not require a hard synchronization. Multiple RA-TDMA rounds for multiple concurrent applications can co-exist in space and time [19]. However, TDMA-based methods typically require slot synchronization, and they are not very dynamic when it comes to changing the beacon period or scheduling retransmissions. Even if they are able to adapt, a high level of coordination and overhead are often required [3,20]. Similarly, retransmissions usually require extra overhead for control data and scheduling, and also a centralized control unit to determine if retransmissions are needed, and when.

In order to compensate for the lack of acknowledgements in the standard, the idea of repetitive broadcast of safety messages in VANETs was proposed so that all vehicle nodes try to rebroadcast the beacon as much as possible to improve packet delivery reliability. For example, in [21], the authors benefit from combining random linear network coding with a repetitive broadcasting scheme. However, these schemes imply quite high complexity and still cannot guarantee reliability.

In contrast to the available literature, our token-based method does not require synchronization nor extra overhead for scheduling of control traffic. Moreover, it is decentralized, can be adapted easily to changes in the beacon frequency, and the amount of redundancy introduced through retransmissions can be adapted based on instantaneous data traffic conditions. Moreover, our protocol can support both beacons and event-driven messages, with high reliability and low delay.

## 3. The Proposed MAC Protocol

A platoon is composed of a leading vehicle and one or more regular platoon members following the leader, each one broadcasting beacons periodically, and event-driven messages whenever needed. One platoon member, the token manager, has special obligations in keeping the token passing protocol running [6]. A one-bit flag is set on all beacons sent by the token manager in order to keep both platoon members and vehicles attempting to join the platoon informed about the token manager’s identity. The token manager is preferably located in the middle of the platoon where radio coverage over the entire platoon is assumed to be best. In this paper, we assumed that the platoon starts from point A, and the platoon formation does not change during the journey to its destination, point B. However, in the real world, the platoon formation can change, and platoon members can join and leave at any time, including the token manager. Then, many questions arise from this, such as: will the token manager role switch from one vehicle to another when the total number of vehicles in a platoon changes dynamically? What mechanism will be used to select the new token manager and perform token manager migration? The new token manager can be selected by voting among platoon members, or can be assigned by the previous token manager.

For practical reasons, e.g., to avoid blocking highway entrances and exits for other vehicles, a platoon is limited in length. It is, therefore, a reasonable assumption that a platoon does not exceed a length of 500 m, which is well below the connectivity range achieved by the 802.11p physical layer. In this paper, the maximum number of platoon members is assumed to be five, similarly to SARTRE [13] (SAfe Road TRains for the Environment)—an EU funded project which ran from 2009 to 2012, and that aimed at developing strategies and technologies to allow vehicle platoons to operate on normal public highways. The privilege to access the channel without competition from other nodes is passed between the platoon members through the token. All messages, both periodic beacons, and event-driven messages, are broadcasted, which enables piggybacking the token on a transmitted message, and notifying all platoon members about the identity of the next token holder, i.e., the vehicle that is allowed to access the channel next. Hereby, the administrative overhead of the token passing protocol is kept low.

### 3.1. Token Passing Operation

Beacons with up-to-date status information are periodically available for transmission at each individual vehicle. It is vital for the control application responsible for managing the safe platoon operation that these status updates are able to access the channel before their content becomes outdated. In order to incorporate data age into the token passing protocol, each vehicle holds a list of all other platoon members and their latest received beacon transmissions. This list is continuously updated as the vehicles listen to activities on the common channel. Whenever a platoon member receives the token, it selects the vehicle on its list with the highest data age, i.e., the oldest reception time stamp, as the next vehicle to transmit its data. Due to the data-age-based selection of the next token holder, our proposed protocol assigns higher priorities to those vehicles that have not been successful in broadcasting their messages for a while. This means that their messages could not be successfully received by all other neighbors in the single hop transmission range. We hereby use the available bandwidth to increase the reception probability of vehicles that temporarily suffer from low connectivity, and to smoothen the delay variations (jitter) throughout the platoon.

**Figure 1:**
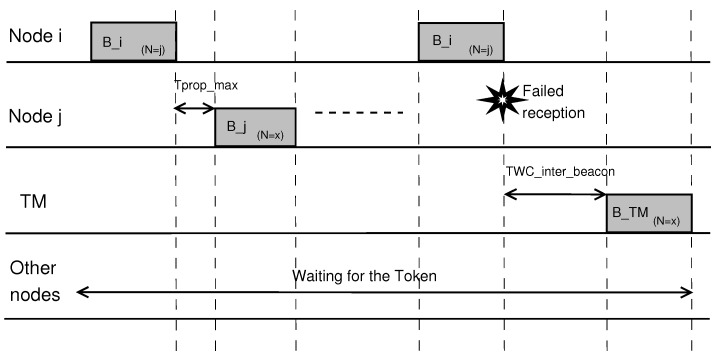
Example of the Token Passing operation and Recovery from a Lost Token.

After receiving a message with the piggybacked token, the platoon member that is selected as the next token holder must wait for a specific period of time (depending on the role of the vehicle in the token passing process and the event-driven transmission method, as explained below) before it can begin its transmission. This waiting period, $T_{waiting}$, is a function of the propagation time, $T_{prop\_max}$, from the first to the last vehicle in the platoon, ensuring that all vehicles had a chance to receive the last transmission before the new token holder takes action. Figure 1 shows an example of our token passing method where node *i* broadcasts its beacon and selects node *j* as the next transmitter. If node *j* receives the beacon from node *i*, it waits for $T_{prop\_max}$ and broadcasts its beacon. Otherwise, the token is lost, and the token manager (TM) must re-generate a new token.

In normal operation, the maximum time between two consecutive tokens amounts to:

(1) $$\begin{array}{c} T_{WC\_inter\_beacon}=T_{trans}+2\times T_{prop\_max}, \end{array}$$

where $T_{trans}$ is the transmission time of a beacon (including a token). As long as the packet is not lost, it requires, in the worst case, an entire $T_{prop\_max}$ to reach its destination, where another $T_{prop\_max}$ is added as a waiting time before channel access is allowed.

### 3.2. Ring Coordination

The token manager is responsible for handling situations that disrupt the normal operation of the token passing protocol. New vehicles joining the platoon have to be integrated into the token passing operation, while vehicles that intend to leave the platoon have to be removed from the protocol in a non-disruptive fashion. Furthermore, considering the unpredictable nature of the wireless channel in vehicular environments, nodes may get temporarily disconnected, and tokens may be lost. The token manager handles these three exceptions as follows:

#### 3.2.1. Temporary Loss of Token

The token manager is the one responsible for generating the first token. If a beacon is lost due to connectivity issues, the token will also be lost as it is piggybacked on the beacon itself. In case of a token loss, the token manager must re-generate the token by (re-)broadcasting its beacon and selecting a new member as the next token holder according to its current member list. Therefore, the token manager monitors the channel and, if it cannot detect any beacon transmission after 3 $T_{prop\_max}$, a new token is inserted into the platoon, and it selects the vehicle with the highest data age as its next target, as shown in Figure 1. In order to avoid a situation where a platoon member that is currently unreachable due to temporary connectivity problems is continuously reselected, the vehicle with the highest data age is only contacted once. If the token is not picked up, upon the next attempt to reintroduce the token, it will be sent to the vehicle with the second highest data age, then to the vehicle with third highest data age, and so forth.

#### 3.2.2. Integration of New Vehicles and Re-Integration of Temporarily Disconnected Vehicles

New vehicles willing to join the platoon have to be integrated into the token passing operation. Furthermore, a chance for temporarily disconnected platoon members to transmit their messages and re-join the protocol has to be provided. As mentioned above, the token manager’s position in the center of the platoon ensures that it has the highest probability to receive beacons from the entire platoon. However, as the platoon length increases, the probability of not being able to hear all platoon members all the time also increases. We consider the case when a transmission from one platoon member cannot be directly received by distant platoon members. In this case, whenever a member fails to receive a beacon from a distant member during one entire beacon period, it removes the member from its local list of platoon members. Note, however, that the removed member would not be removed from the local lists of all members, and thus it will eventually be chosen as the next token holder by a nearby vehicle, thereby remaining in the token loop.

A more serious problem occurs when one member is removed from the local lists of all other platoon members, including the token manager. In that rare but theoretically possible case, the removed member will be totally disconnected from the platoon. In order to allow this vehicle to rejoin the platoon and receive the token again, we introduce a joining phase where vehicles (both new vehicles who want to join, and those who suffered from complete disconnection) get a chance to send their packets and join. In our experiments, we assumed that members will try to rejoin the platoon and receive the token again via the joining phase after two entire beacon periods. However, this number can be tuned based on the number of platoon members and application requirements.

**Figure 2:**
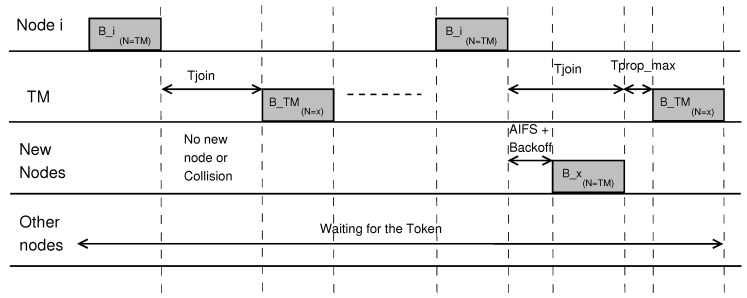
Integration of new vehicles.

The token manager will, each time it receives the token, wait for a period $T_{join}$ until it is allowed to send its beacon. During $T_{join}$, vehicles compete for channel access according to the IEEE 802.11p-compliant CSMA random access protocol. Therefore, the length of $T_{join}$ depends on the propagation time, the data packet length, and the maximum back-off time:

(2) $$\begin{array}{c} T_{join}=T_{trans\_join\_request}+T_{AIFS}+T_{backoff\_max}+T_{prop\_max}, \end{array}$$

where $T_{trans\_join\_request}$ is the length of the data packet carrying the request to join the platoon. A vehicle listening to the channel hears that the token manager is selected as the next token holder and attempts to access the channel according to the CSMA rules, i.e., it listens to the channel for an Arbitrary Inter Frame Spacing (AIFS) period plus a random additional time; if the channel remains idle, it sends its packet. In case of a packet collision, it must wait to detect the next joining period, as we assume that only one node can join the group in each period. $T_{join}$ denotes the maximum time needed to accommodate one successful channel access. The token manager finishes the joining period by resuming its beacon transmission, or upon receiving a packet from a new member. As shown in Figure 2, node *i* broadcasts its beacon and sends the token to the token manager. The token manager delays its transmission, thereby allowing all nodes that are waiting to join the platoon to do it. If there is no joining candidate, or when a collision occurs, no one will actually join and the bandwidth is wasted. In the next joining phase, node *x* wins the competition after waiting for a period of time equal to AIFS plus backoff, and broadcasts its beacon. The token manager resumes its transmission after $T_{prop\_max}$.

#### 3.2.3. Removing Vehicles from the Token Loop

Vehicles will be removed from a local member list after a time of inactivity, denoted as $T_{inactive}$,

(3) $$\begin{array}{c} T_{inactive}=N\times T_{WC\_inter\_beacon}, \end{array}$$

where $T_{WC\_inter\_beacon}$ is the maximum delay between two consecutive beacons according to Equation (1) above, and *N* is the number of vehicles in the platoon. A vehicle with short-term connectivity problems to far-away platoon members will be temporarily removed from a few local lists, but, eventually, it will be re-added as long as its presence remains known to some platoon members. A vehicle that intentionally leaves the platoon will eventually disappear from all local lists.

In the IEEE 802.11p standard [8], there is no retransmission scheme for unsuccessful broadcast transmissions since there is no way to determine if reception was successful. Merely increasing the beacon update rate might just add to the problem by increasing the probability of packet collisions, lowering the performance even further [4]. Our method actually proposes a built-in retransmission scheme as platoon members search in their lists and select a member based on beacon age to pass the token to. The algorithm just keeps selecting nodes with the highest data age, thereby automatically offering retransmission opportunities to those nodes that had no success for a while. This way, the transmitter side does not need any mechanisms, such as acknowledgements, to guarantee a successful reception at the receiver side. Therefore, the number of retransmissions is dynamically selected based on the current channel conditions. Our protocol also introduces a more flexible and scalable scheduling mechanism compared to TDMA-based schemes for VANETs, and specifically for platooning applications. Due to the distributed nature of the protocol, members independently manage beacon transmissions; this way, the protocol is able to automatically adapt itself to changes in the network scenario, such as the platoon size or the beacon generation frequency. A pre-scheduled TDMA-based retransmission scheme is, on the other hand, much more static, requiring rescheduling and control data exchanges to adapt to changes.

### 3.3. Integration of Event-Driven Messages

We distinguish between two groups of event-driven messages. The first type is made up of highly time-critical warning messages, as e.g., the information that needs to be spread throughout the platoon with minimal delay to react to sudden and unforeseen breaks or collision avoidance manoeuvres. The second type is less time critical. It addresses the sporadic distribution of maintenance information, and generally originates from the platoon leader. Different event-driven message types require different services. Hence, we propose three methods to transmit event-driven messages while maintaining token-based beacon broadcasting, including dedicated phase for event-driven messages, event-driven message transmission upon token reception, and event-driven message transmission without token. Note that the token is only passed on with a beacon in the dedicated phase for event-driven messages and the event-driven transmission upon token reception methods, while, in the event-driven transmission without a token method, the token is piggybacked on both beacon and event-driven messages.

#### 3.3.1. Dedicated Phase for Event-Driven Messages

The joining phase for the integration of new vehicles into the token loop is extended to provide room for event-driven messages. Every time the token manager holds the token, it will delay its beacon transmission to give platoon members an opportunity to send their event-driven messages. As in the joining phase, channel access is determined in competition with other vehicles using the standard-compliant CSMA MAC protocol. In order to increase the probability that the event-driven message is received by all vehicles, it should be repeated by each vehicle upon reception. A long CSMA-phase ensures that the event will spread throughout the platoon in a multi-hop fashion before the phase is over. On the other hand, the token passing process, and thereby the periodic dissemination of beacons, will be delayed. Furthermore, if there are no events to be reported, a considerable amount of valuable bandwidth will remain unexploited, wasting valuable resources. The occurrence of the event phase depends on how often the token manager receives the token. For long platoons, this might introduce a considerable delay, especially if the event happened right after the token manager passed on its token, and if spreading the event message throughout the platoon requires more than one event phase.

#### 3.3.2. Event-Driven Transmission upon Token Reception

Here, a vehicle sends its event-driven data whenever it receives the token. Event-driven packets and beacons are kept in separate queues and, when a vehicle holds the token, packets in the event queue get higher priority than beacons. Only when all its event-driven data are sent does the vehicle send out its beacon, attaching the token to the next token holder. Note that the token is only passed on with a beacon, not with an event-driven message. This method needs no bandwidth reservation. An event might, however, happen right after the vehicle passed on its token, meaning that it has to wait for a long time until it gets the token again, adding unwanted delay. Furthermore, considering the need for relaying event-driven data to increase the probability of successful delivery, every node that receives the event-driven message from another vehicle has to wait for the token before the message can be passed on. This adds a delay between the relaying opportunities, and leads to a much longer dissemination delay compared to the first method, where the entire dissemination can be done within the duration of the reserved phase.

#### 3.3.3. Event-Driven Transmission without Token

For this approach, we introduce different waiting periods for token passing and event-driven message transmissions, respectively. Vehicles with event-driven messages are allowed to be more opportunistic than the current token holder. Once a beacon (and therefore token) is received, a countdown is started at the new token holder. While the waiting time ($T_{waiting}$) is equal to one maximum propagation time ($T_{prop\_max}$) when no event-driven traffic is considered (see Section 3.1), we increase $T_{waiting\_token}$, and thereby provide vehicles with event-driven data an opportunity to seize the channel before the token holder does. When the token holder checks the channel after $T_{waiting\_token}$, it will find the channel busy because of an event-driven message transmission, and so it cancels its own transmission. In contrast to the two previous methods, the token is sent along with the event-driven message. Therefore, the previously selected token holder loses its current transmission opportunity, but has an opportunity to be selected again for data transmission by the event-driven message transmitter.

### 3.4. Multi-Hop Dissemination Method

Due to channel problems, packets from the platoon leader may not be received by a vehicle at the back of the platoon. In those cases, a dissemination strategy is needed. Specifically, intermediate vehicles who successfully received the event-driven messages will relay this information to the back of the platoon. In this paper, we choose the simplest dissemination strategy for this purpose, which is flooding with one repetition. Therefore, upon receiving an event-driven message, a vehicle keeps the message in its relaying table and re-broadcasts the message as soon as it gets channel access. We assign the highest priority to relayed messages so that all available messages in the relaying table must be transmitted. Afterwards, the vehicle is allowed to send its own messages.

## 4. Numerical Analysis

In this section, we will present numerical analysis for our proposed methods, including methods for beacon (when we assume there is no event-driven message transmission) and event-driven message transmission. In our analysis, for simplicity and to show the upper bound for channel access delay, we assumed worst-case examples.

### 4.1. For Beacon Transmissions (without Event-Driven Traffic)

Equation (1) in Section 3.1 showed the maximum beacon inter-arrival time, without accounting for any joining period, lost token, or a phase for event-driven messages. A join period is an extra period during which joining is allowed, and it takes place after the manager gets the token (see Equation (2)). Therefore, for *N* vehicles, the worst-case beacon round-trip time when no packets are lost is:

(4) $$T_{WCbeaconRT}=N\times T_{WC\_inter\_beacon}+T_{join},$$

where $T_{join}$ is added for the one occasion during the round-trip when the manager holds the token. Note that if, e.g., the last vehicle in the platoon loses a message from the token manager, the manager may get the token more frequently, and thus the join period will occur more frequently. Equation (2) therefore represents the typical case in a fully connected platoon, where all platoon members have the same chance of getting the token.

### 4.2. For Event-Driven Traffic

#### 4.2.1. Dedicated Phase for Event-Driven Messages

We assume that join requests and event messages compete with each other in an event/join phase right after the token manager gets the token. Only one message gets channel access, either the event message or the join request, and event-driven messages get higher priority than join requests. The time for the event/join phase would be:

(5) $$\begin{array}{c} T_{eventjoin}=max\left(T_{trans\_event},T_{trans\_join\_request}\right)+T_{AIFS}+T_{backoff\_max}+T_{prop\_max}. \end{array}$$

To calculate the worst case waiting time until an event message can access the channel, we consider the worst-case situation where an event is detected during an on-going event/join phase, but too late to accommodate a packet transmission. The event packet would then have to wait for

(6) $$\begin{array}{c} T_{WC\_event\_waiting\_time}=max\left(T_{trans\_event},T_{trans\_join\_request}\right)+N\times T_{WC\_inter\_beacon}+T_{AIFS}+T_{backoff\_max}, \end{array}$$

i.e., the time until the current event/join message is sent, plus the full token round-trip time until the manager gets the token again, plus the worst case time to get CSMA channel access in the next event/join phase.

#### 4.2.2. Event-Driven Message Transmission after Token Reception

Here, the worst case beacon round trip time must be extended to the case where every vehicle has an event message to send every time it gets the token:

(7) $$\begin{array}{c} T_{WC\_inter\_beacon\_event}=T_{trans\_event}+T_{trans\_beacon}+2\times T_{prop\_max}, \end{array}$$

(8) $$\begin{array}{c} T_{WCbeaconRT\_event}=N\times T_{WC\_inter\_beacon\_event}+T_{join}. \end{array}$$

If we assume that a single retransmission for each message in the relaying table is required to reach all platoon members, we have to add the worst-case waiting time where all vehicles already have *N*-1 messages in their relaying table. Therefore, they have to send all packets in their tables before sending their own packets, and this adds more delay to the $T_{WC\_inter\_beacon\_event}$ as follows:

(9) $$\begin{array}{c} T_{WC\_inter\_beacon\_event\_relay}=T_{trans\_event}+T_{trans\_beacon}+\left(N-1\right)\times T_{trans\_event}+2\times T_{prop\_max}. \end{array}$$

#### 4.2.3. Event-Driven Message Transmission without Token

Here, we extend the $T_{waiting\_token}$ to $2\times T_{prop\_max}$ in order to offer any vehicle with a pending event the chance to seize the channel after a waiting time of $T_{waiting\_event}$ lasting for one $T_{prop\_max}$ plus a random backoff time. This changes all the worst-case calculations for beacon transmissions under Section 3.1.

The event, in the worst case, is detected just when another vehicle obtains the token, and therefore our vehicle has to wait until the next time the token is passed to opportunistically seize the channel. In the worst case, the vehicle that just got the token is the token manager, and so we have to add the joining phase:

(10) $$\begin{array}{c} T_{WC\_event\_waiting\_time}=T_{trans\_beacon}+T_{prop\_max}+T_{waiting\_event}+T_{join}+T_{backoff\_max}. \end{array}$$

## 5. Simulation Settings

To assess the effectiveness of the proposed protocol, we rely on computer simulations, where we use the analytical evaluation from Section 4 to verify the simulator. In this section, we describe the simulation details, including simulation scenario parameters and protocol configurations.

We simulate platoons of five vehicles on a highway, a setting commonly used for platooning applications [22], with an antenna-to-antenna spacing of 30 m. We used SUMO (0.17.0) [23] in order to generate realistic vehicular mobility models. In addition, we implemented our proposed MAC protocols in OMNeT++ (version 4.4.1) [24], and used the IEEE 802.11p implementation made available by the Veins framework (version 2.1) [25] for OMNeT++ for performance comparison purposes. Table 1 and Table 2 summarize the simulation and protocol parameters.

According to the ETSI standard, we use 400 byte packet sizes for beacon and event-driven messages. Beacons and event-driven messages are generated every 20 ms and 50 ms, respectively. In addition, packets are broadcasted with a data rate of 6 Mbps within a transmission range of 500 m. Similarly to [26], we combine simple path loss and log-normal shadowing models, which are common models for highway simulation. In addition, the parameters of these models were chosen according to that same paper. We ran each simulation for 20 min, and repeated them 300 times. The beacon and event-driven priorities are AC[0] and AC[1], respectively.

The metrics used to evaluate the performance of the MAC protocols are summarized as follows: (a) Packet Delivery Ratio (PDR) of event-driven messages, which represents the ratio of the total number of packets received by the final destinations and the packets originated by the source; (b) average channel access delay of event-driven messages, which represents the average time for a packet to wait before access to the channel is granted; and (c) Inter-Reception Time (IRT) of periodic beacons, which is calculated as the time interval between the sequential reception of beacons from each member averaged over all platoon members. The IRT parameter reflects the data age of the beacon content as it monitors the age of the information a node holds from a specific neighbor once a new beacon arrives. Maintaining an IRT close to the beacon period is vital to the successful implementation of a platoon control application.

## 6. Simulation Results and Analysis

In order to show the advantages of the proposed protocol, we evaluate the performance of our proposed protocol and methods under both single and multi-hop broadcasting scenarios. Since this paper is mainly focused on single-hop broadcasting, we first show a detailed analysis for single-hop broadcasting; then, we evaluate the best performing single-hop solutions, in terms of reliability and delay, and their ability to support multi-hop broadcasting. IEEE 802.11p is used as a reference, and we compare the delay and the reliability of our protocol for supporting beacons and event-driven messages.

### 6.1. Single-Hop Broadcasting

**Figure 3:**
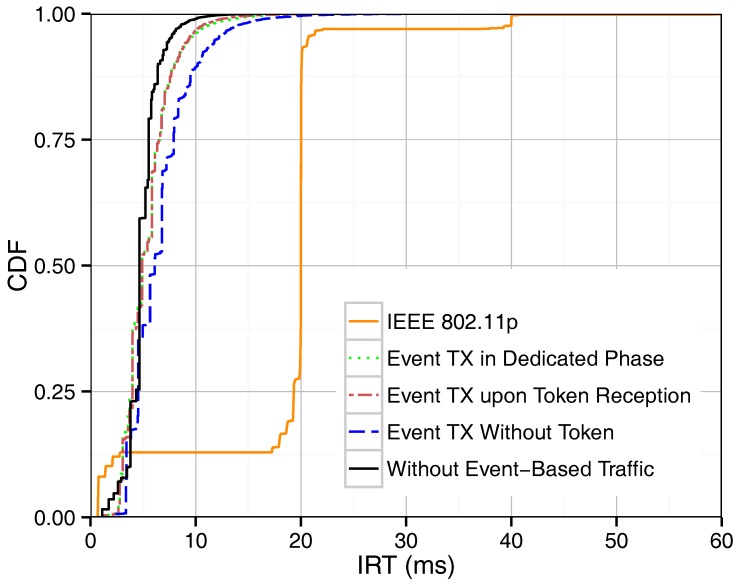
CDF (Cumulative Distribution Function) of IRT (Inter-Reception Time) for single-hop broadcasting.

As mentioned above, we proposed three different methods for supporting event-driven messages. Figure 3 shows the IRT of beacons in our token-based MAC protocol with and without the presence of event-driven traffic in the network, and it provides a performance comparison with IEEE 802.11p. In the case of IEEE 802.11p, beacons and event-driven traffic attempt to access the channel only once per assigned period. The IRT of IEEE 802.11p beacons monitored in Figure 3 lies therefore mostly around the 20 ms mark, which is the beacon period defined. Delayed channel access due to contention leads to beacon IRTs that are longer or shorter than exactly one period, as shown in the graph for IEEE 802.11p. The proposed token passing scheme, on the other hand, retransmits beacons within one period as long as there are resources available. Therefore, considerably shorter IRT values are achieved. As expected, the case where no event-driven traffic is present in the network is associated to the lowest IRT values. The introduction of event-based traffic, regardless of the method chosen, utilizes bandwidth for event-based packets instead of beacon retransmissions, and therefore shows a slight increase of IRTs. If event-driven messages are sent during a dedicated phase or upon token reception, they simply occupy the bandwidth that would otherwise be used for beacon retransmissions, and thus they have only a small influence on the IRT. On the contrary, the third method, where event-based messages can be sent without waiting for the token, actually interferes with the token passing order, resulting in decreased beacon performance. Using Equation (4), the maximum round-trip time without considering token losses is equal to 9.4 ms. As shown in the figure, the maximum IRT value is in general about 10 ms, being higher values related to token losses.

**Figure 4:**
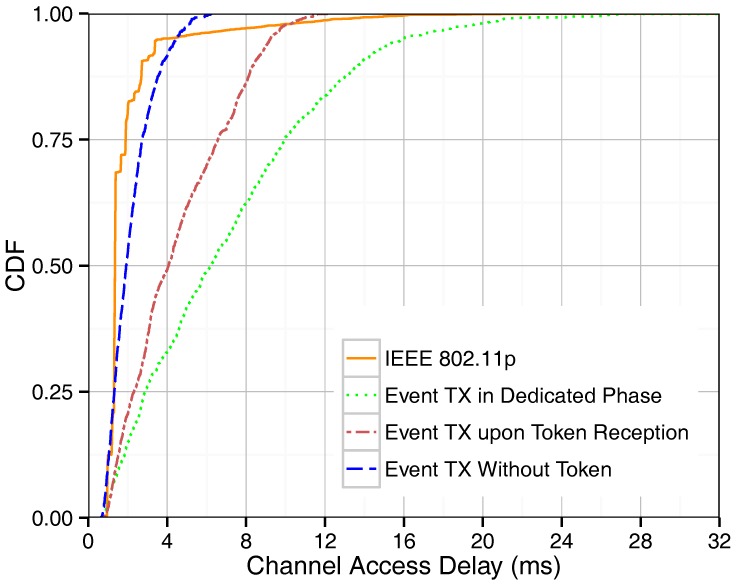
CDF of channel access delay for single-hop broadcasting.

The channel access delay of event-driven messages for IEEE 802.11p and our proposed methods are shown in Figure 4. As expected, IEEE 802.11p obtains shorter delays for event-driven messages since it uses a random access method and, therefore, event-driven messages are sent as soon as the channel is found to be idle. The opportunistic fashion of transmitting event-based packets without token possession shows the best performance, as it allows time-critical event-based traffic to access the channel as soon as the need arises. The delay increases if an event-driven message generated by a certain vehicle has to wait until that specific vehicle happens to get the token before it can be sent, as occurs in the “event tx upon token reception” method. The delay is even longer when a vehicle has to wait for the event phase in order to send its event packet (“event tx in dedicated phase”). This phase is then shared by all event-driven messages generated by different platoon members since the last event phase took place, leading to contention and further delays. The “event tx without token” achieves channel access delay results close to those of IEEE 802.11p since the event-driven messages are given a higher priority than beacons, and also because the normal beacon transmission routine is interrupted to provide room for event-driven message transmission. Based on the Equations (6), (8) and (10), the channel access time for event-driven messages for “event tx in dedicated phase”, “event tx upon token reception”, and “event tx without token” methods are 9, 11.8, and 3.1 ms, respectively. Figure 4 shows that the simulation results are only compliant with the numerical results for “event tx upon token reception” method since, in this method, the event-driven transmission routine does not interfere with the token passing routine. Concerning the other methods proposed, the number of collisions increases since event-driven message transmissions interfere with the beacon transmission routine, which is not considered by the numerical analysis.

Although IEEE 802.11p obtains a lower channel access delay, notice that the delivery ratio for event-driven messages achieved by our protocol is higher than for 802.11p, as shown in Table 3. The results show that waiting for the token gives the best delivery ratio since it does not interfere with the normal beacon transmission routine for event-driven messages, which leads to fewer collisions. In addition, introducing a dedicated phase for event-driven traffic once per token period represents the worst case among our proposed methods since, for this solution, all platoon members must compete with each other in each such period in order to send their event-driven messages, and they must compete with join requests from non-platoon members as well. Moreover, the table shows that the delivery ratio distribution for all platoon members is almost uniform since collisions are fewer as nodes become synchronized with the token passing procedure by taking the propagation delay into account.

Overall, simulation results show that the “event tx upon token reception” method is best when transmission reliability is the most important factor, while the “event tx without token” method is preferable for time-critical warning messages due to its lower channel access delay. Furthermore, keeping the beacon data age-based token selection intact reduces the negative impact of event-driven traffic on the beacon performance. This should be considered in favor of the “event tx upon token reception” method.

### 6.2. Multi-Hop Broadcasting

As we mentioned above, the main focus of our proposed protocol is single-hop broadcasting. Nevertheless, in order to increase the probability that all platoon members successfully receive event-driven messages, we extend our protocol to enable multi-hop message delivery. By introducing multi-hop relaying features, each event-driven message is rebroadcasted once upon reception. This way, the chances of reaching all platoon members with information about a specific event increase. With the exception of the “event tx in dedicated phase” method, which shows the worse performance in terms of event-driven message delivery and channel access delay for single-hop broadcasting, we have extended all methods with multi-hop features for further comparison.

**Figure 5:**
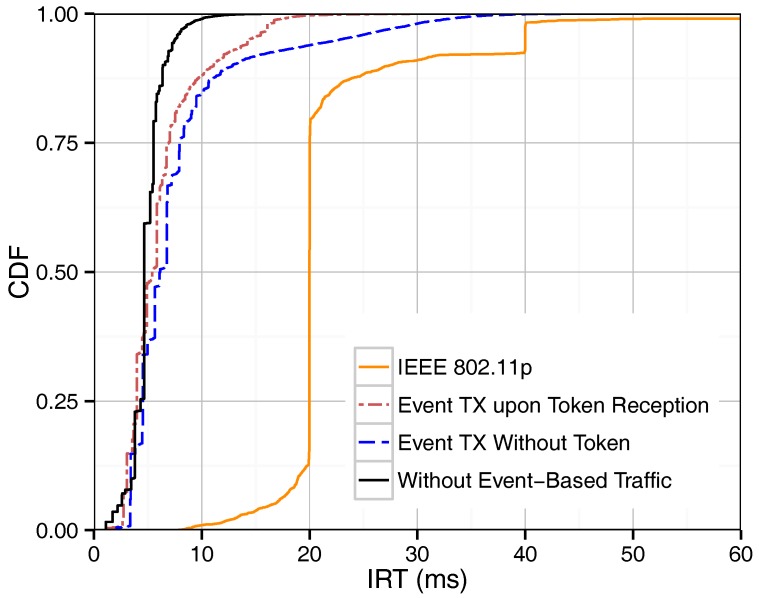
CDF of IRT for multi-hop broadcasting.

Figure 5 shows the IRTs for IEEE 802.11p and our protocol. As shown, the performance levels achieved by the “event tx upon token reception” method are quite close to those achieved without event-driven traffic. Notice that, since event-driven message transmission in the “event tx upon token reception” method does not interfere with the normal beacon routine, IRTs for single and multi-hop broadcasting show a very similar trend. In contrast, the performance of the “event tx without token” method is degraded since this method interferes with the token passing to broadcast event-driven and forwarding messages, thereby causing several collisions and increasing IRT values. Compared to the single hop case, IEEE 802.11p performance decreases when introducing multi-hop broadcasting due to the increased traffic volume.

**Figure 6:**
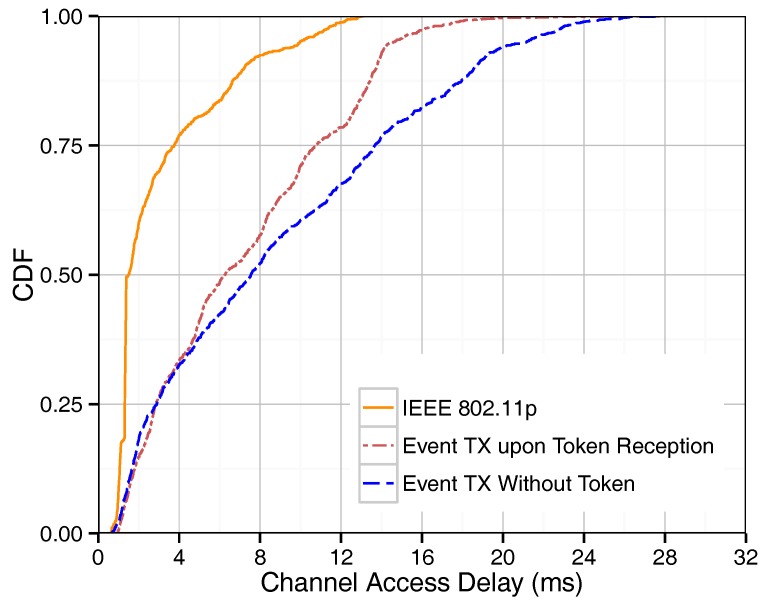
CDF of channel access delay for multi-hop broadcasting.

Figure 6 shows the channel access delay for IEEE 802.11p and our protocols. As expected, IEEE 802.11p achieves better performance compared to our methods in terms of delay since 802.11p allows immediate access to the channel as soon as it is idle. Instead, for the “event tx upon token reception” method, a vehicle carrying an event-driven message to send must wait for the token before transmitting or relaying. However, the maximum delay achieved by our protocol never exceeds 20 ms, thereby meeting the required delay bound for safety applications [27]. In contrast to single-hop broadcasting, the “event tx without token” method shows a longer channel access delay than the “event tx upon token reception” method due to the high traffic load by forwarding messages. In the “event tx without token” method, after any event-driven message reception, all platoon members attempt to forward such message using CSMA much like with IEEE 802.11p. Therefore, the probability of collision is increased, which also leads to increased channel access time.

Complementing Figure 6, Table 4 shows that, although our protocol obtains higher channel access delays than 802.11p, it has better delivery ratios, so that the “event tx upon token reception” method successfully delivers all event-driven messages with only one rebroadcast of each event-driven message received. Therefore, our protocol is able to successfully deliver event-driven packets within the deadline for safety applications while, at the same time, maintaining a beacon delivery effectiveness of at least one beacon every beacon interval.

## 7. Conclusions

MAC layer solutions able to provide high reliability and bounded channel access delays are of utmost importance to deploy critical services over VANETs. In this paper, we proposed an enhancement to our previously-designed token-based MAC protocol, providing timely and reliable inter-vehicle communication for beacon and event-driven messages. The proposed protocol has the following features: (a) the protocol uses a token to achieve non-competitive channel access; (b) it automatically provides retransmission opportunities for platoon members based on data age; (c) it is able to integrate new vehicles into the platoon and remove those vehicles that intend to leave the platoon in a non-disruptive fashion; and (d) three different extensions to the protocol were proposed to support different types of event-driven messages, along with beacon messages.

Analytical and simulation-based evaluations have been provided for single and multi-hop broadcasting scenarios. The results show, on the one hand, that the proposed method is able to fulfill the requirements for beacon transmission and guarantee beacon delivery within one beacon generation interval. On the other hand, it shows that different methods for disseminating event-driven messages are useful for different types of events, thereby being able to meet the requirements of a wide variety of safety applications. The proposed protocol clearly outperforms IEEE 802.11p for beacon transmission in single and multi-hop scenarios. In addition, it shows a better delivery ratio and limited delay degradation for event-driven messages. In addition, our token-based method is decentralized, not requiring synchronization nor any extra overhead for control traffic, thereby adapting itself easily to changes in the beacon frequency and the number of platoon members.

## Figures and Tables

**Table 1:** The simulation parameters.

Simulation Parameter	Value
Simulation time	20 min
Platoon size	5 vehicles
Propagation model	Simple path loss + Log-normal shadowing
Antenna-antenna spacing	30 m
Frequency	5.9 GHz
Beacon frequency	50 Hz
Beacon length	400 bytes
Event-driven message frequency	20 Hz
Event-driven message length	400 bytes
Data Rate	6 Mbps
Transmission range	500 m
Time slot	13 μs
SIFS time	32 μs

**Table 2:** The protocol parameters.

Protocol Parameter	Value
$T_{waiting}$ ($T_{prop\_max}$)	0.5 ms
$T_{waiting\_event}$	0.5 ms
$T_{waiting\_token}$	1 ms

**Table 3:** Average Event-Driven Message Delivery Ratio for single-hop broadcasting.

Average Event-Driven Message Delivery Ratio (%)				
*Vehicle ID*	*IEEE802.11p*	*Event TX in Dedicated Phase*	*Event TX upon Token Reception*	*Event TX without Token*
1	77.00	87.00	95.25	88.25
2	84.00	87.00	96.25	87.25
3	85.00	87.00	97.00	87.00
4	83.00	87.00	96.50	88.25
5	77.00	87.00	96.00	89.87
**Average**	**81.20**	**87.00**	**96.20**	**88.14**

**Table 4:** Average Event-Driven Message Delivery Ratio for multi-hop broadcasting.

Average Event-Driven Message Delivery Ratio (%)			
*Vehicle ID*	*IEEE802.11p*	*Event TX upon Token Reception*	*Event TX without Token*
1	92.75	100	94.00
2	87.25	100	91.50
3	89.50	100	90.75
4	90.00	100	91.00
5	87.25	100	94.50
**Average**	**89.35**	**100**	**92.35**
